# Exposure Assessment for Italian Population Groups to Deoxynivalenol Deriving from Pasta Consumption

**DOI:** 10.3390/toxins5122293

**Published:** 2013-11-26

**Authors:** Carlo Brera, Valentina Bertazzoni, Francesca Debegnach, Emanuela Gregori, Elisabetta Prantera, Barbara De Santis

**Affiliations:** Istituto Superiore di Sanità, Dipartimento di Sanità Pubblica Veterinaria e Sicurezza Alimentare, Reparto OGM e Xenobiotici di origine fungina, Viale Regina Elena, Rome 299-00161, Italy; E-Mails: bertazzonivale@gmail.com (V.B.); francesca.debegnach@iss.it (F.D.); emanuela.gregori@iss.it (E.G.); elisabetta.prantera@gmail.com (E.P.); barbara.desantis@iss.it (B.D.S.)

**Keywords:** deoxynivalenol, pasta, exposure assessment, risk assessment, consumer groups, children, cereals

## Abstract

Four hundred and seventy-two pasta samples were collected from long retail distribution chain sales points located in North, Central and South Italy. Representative criteria in the sample collection were followed in terms of number of samples collected, market share, and types of pasta. Samples were analysed by an accredited HPLC-UV method of analysis. The mean contamination level (64.8 μg/kg) of deoxynivalenol (DON) was in the 95th percentile (239 μg/kg) and 99th percentile (337 μg/kg), far below the legal limit (750 μg/kg) set by Regulation EC/1126/2007, accounting for about one tenth, one third and half the legal limit, respectively. Ninety-nine percent of samples fell below half the legal limit. On the basis of the obtained occurrence levels and considering the consumption rates reported by the Italian official database, no health concern was assessed for all consumer groups, being that exposure was far below the Tolerable Daily Intake (TDI) of 1000 ng/kg b.w/day. Nevertheless, despite this, particular attention should be devoted to the exposure to DON by high consumers, such as children aged 3–5 years, who could reach the TDI even with very low levels of DON contamination.

## 1. Introduction

Deoxynivalenol (DON) is a natural-occurring mycotoxin produced at pre-harvest stage by several *Fusarium* species, mainly *F.graminearum* and *F. culmorum* [1], and belongs to a wide family of mycotoxins known as trichothecenes. It is also known as vomitoxin due to its strong emetic effects after consumption, because it is transported into the brain where it runs dopaminergic receptors.

**Figure 1:**
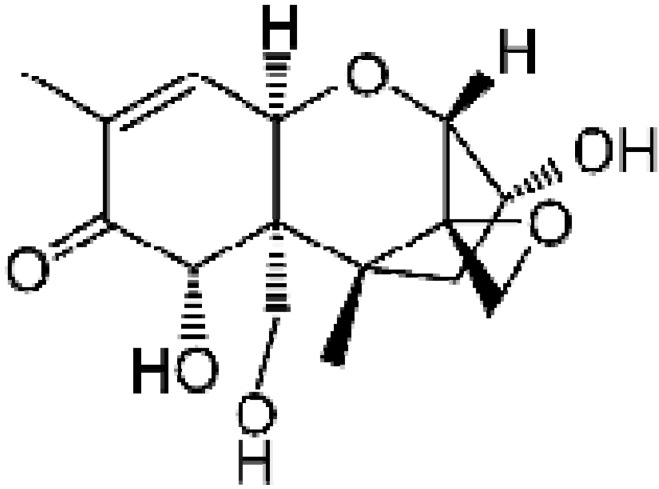
Chemical structure of deoxynivalenol (DON).

Chemically, DON (Figure 1) is a sesquiterpenoid polar organic compound, which belongs to the type B trichothecenes since it contains carbonyl group in C-8. Its empirical formula is C_15_H_20_O_6_. DON is highly hydrosoluble and stable at cooking temperatures (120 °C), and in storage conditions and milling processes [2,3].

DON is one of the most pervasive mycotoxins that predominantly colonize wheat ears and also corn leaves. Fungi attack mainly occurs in the field before harvest [4]. 

The optimal range of temperatures for DON production is 21–29 °C at moisture levels >20%. DON is considered as a marker for the presence of other mycotoxins such as zearalenone [5]. 

The fungus has two distinct growth cycles corresponding to mould growth during warm daytime temperatures and toxin production during cool night-time temperatures [6].

Red ear rot caused by *F. graminearum* is favoured by warm wet weather after silking. This plant disease tends to be more risky in conditions of no rotation between subsequent cultivations, and in general when corn or wheat precedes wheat and corn crops respectively as a consequence of the permanence of contamination in the debris. Reduced tillage situations, provides additional elements for an increase of the probability of fungi attack.

To date, all animal species are susceptible to DON in the following order: pigs > mice > rats > poultry ≈ ruminants [7]. Differences in metabolism, absorption, distribution, and elimination of DON among animal species might account for this differential sensitivity.

Specifically in swine, DON intake reduces weight gain and hinders animal feeding. At high concentrations (more than 10 ppm) typical signs are emesis and total feed refusal [8]. In terms of DON bioavailability, sheep and cows show very low rates (10% for single-doses administered) [9] in contrast with swine where approximately 95% of the administered dose was recovered as deoxynivalenol [10].

Acute exposure of pigs to DON causes abdominal distress, increased salivation, malaise, diarrhea, and emesis [11,12].

DON is detectable also in blood and serum in high amounts immediately after ingestion, but is rapidly cleared from the blood stream.

Although JECFA established that DON is a probable factor for acute pathologies in humans, there is not enough data yet to set an Acute Reference Dose (RfD) [13]. 

In 1993, IARC classified DON in Group 3, corresponding to not classifiable for its carcinogenicity to humans; in 2002 the Scientific Committee for Food set a tolerable daily intake of 1 μg/kg bw/d. 

In humans, deoxynivalenol causes gastro-intestinal problems, immunosuppression and interferences with reproduction and development [14,15,16,17,18]. DON effects in humans have not yet been widely registered but new evidence that has led to a report of immunotoxicity in humans even at low doses of contamination that could create proteomic changes in human B (RPMI1788) and T (JurkatE6.1) lymphocyte cell lines is currently under consideration. These potential effects are to be considered much more alarming if transferred to the fetus where, according to a Norwegian study, 21% of the toxin is transferred, with no activation of DON required and a very poor detoxification route. More generally, a correlation between low sanitary quality of cereals and increase of abortions during pregnancy was also noted [19,20,21].

*In vitro* studies using human intestinal Caco-2 cells suggested that DON crosses the intestinal mucosa through a para-cellular pathway, though contribution by passive trans-cellular diffusion could not be ruled out [17,22].

From the above, this study provides an evaluation of the exposure to deoxynivalenol deriving from the consumption of pasta by different groups of Italian consumers, with a focus to a very sensitive subgroup such as children. 

### 1.1. Legislation on DON

Almost 40 countries have established regulatory limits or guidelines for DON in wheat and other cereal-based products. The guideline levels for cereals and finished cereal products for humans range from 100 to 2000 μg/kg, depending on consumer age and stage of processing of the grain [23]; levels in diets for swine, poultry, and cattle range from 500 to10.000 μg/kg [24]. 

The European Commission set maximum tolerable limits for DON in food products. EC Regulation 1126/2007 applies to the unprocessed durum wheat and oat (1750 μg/kg), soft wheat (1250 μg/kg) and to milled intermediate products, e.g finished products (500 μg/kg), dry pasta, cereals destined for direct human consumption, such as cereal flour, bran and germ (750 μg/kg) and processed cereal-based baby foods and foods for young children (200 μg/kg) [25]. Currently, no legal limits but only guidance values have been set by the EU Recommendation 576/2006 for complementary and complete feeding stuffs at various levels depending on the animal species susceptibility such as pigs, calves (<4 months), lambs and kids [26].

However, it should be considered that DON contribution to animal origin food products as carried over from feeds is generally negligible since no residues in eggs, milk and edible tissues were found in the literature. It was shown, in fact, that DON is rapidly metabolized by de-epoxydation and glucuronization leading to the formation of reduced toxicity metabolites [27].

### 1.2. Exposure Assessment by DON in Humans

Generally, human exposure assessment derived from mycotoxin-contaminated diet is a more and more challenging issue in the worldwide scenario. What is still pending is the real aetiological role of these hazards in the development of pathologies such as cancer or mycotoxin-induced immunodepression diseases or other pathologies not yet related, like autism or celiac disease [28,29]. 

From the data available in the literature, in humans, the emetic effects of this mycotoxin were firstly described in Japanese men consuming mouldy barley containing *Fusarium* fungi in 1972 [30,31]. 

In China, between 1961 and 1985, about 35 outbreaks of acute human illness were reported. The symptoms of nausea, vomiting, diarrhea, abdominal pain, headache, dizziness, and fever were attributed to DON and other trichothecenes contaminated cereals, with at least 7818 victims. 

In an outbreak in 1984 in Xingtai County, 94% persons who ate moldy maize became ill. The range of DON levels was from 3.8 to 93 mg/kg [13].

DON was detected in all 15 urine samples of female inhabitants of Linxian County and Gejiu, two Chinese high and low, respectively, risk exposure regions for DON and oesophageal cancer, with mean levels of 37 and 12 ng/mL, respectively [32].

In one-year-old Dutch children exposed to DON levels above the Provisional Maximum Tolerable Daily Intake (PMTDI), reductions in body weight and relative liver weight were estimated at 2.2% and 2.7% (confidence interval: 0.2%–25%), respectively [33].

In a study performed in UK, DON was detected in the urine of 296 out of 300 healthy subjects showing a strong association between the cereal intake and urinary DON concentrations (*p* < 0.0005). From a multivariable analysis, wholemeal and white bread as well as other cereal-based food products including pasta were consistently related to urinary DON excretion. 

The geometric mean concentrations were 6.55, 9.63, and 13.24 μg DON/day for low-, medium-, and high-cereal intake groups, respectively. Consumption of other grain-based foods such as cereal products and pasta was also significantly associated with urinary DON concentrations [34].

In another study by Hepworth *et al.*, DON exposure assessment was evaluated in a group of pregnant women aged 16–44 from Bradford, UK. The urinary DON was detected in all samples in a range from 0.5 to 116.7 ng/mg creatinine. From a food questionnaire, bread, particularly chapattis in South Asian women, was the major contributor to DON exposure [35].

In 2003, the study performed by the European Commission within the SCOOP task 3.2.10 revealed that the total intake of DON calculated from data coming from 12 member States ranged from 14.45%–46.1% to 11.3%–95.9% of TDI for adults and children, respectively. The contribution deriving from wheat and derived products accounted for 76%–90%. In this study, among foodstuffs, pasta showed a lower rate in the overall contribution to DON intake (25%) compared to wheat flour and bread [36].

Analogous results were obtained by Larsen *et al.* [37] where higher intakes were calculated considering the 95th percentile of consumption data multiplied by the mean DON concentration resulted in an intake very close to or even higher than TDI, with specific emphasis for children and infants. 

It should be noted that in another study performed on baby foods in Italy by Pietri *et al.* in 2004, DON intakes higher than the TDI (121%) were observed. In this study, a consumption of 100 grams of cereal-based products was considered [38]. 

#### Occurrence of DON in Wheat Products

So far, in order to estimate the real amount of DON ingested with human diet, a reliable assessment should be made, taking into account (i) the metabolic pathways of DON leading to the formation of various DON-metabolites, DOM-1, glucuronic-DON, 3-AcOH-DON and 15-AcOH-DON; (ii) the lifecycle of DON from the raw kernel to the finished products such as flour or bread and pasta; and (iii) the frequency and degree of the occurrence of DON contamination levels in the wheat products ingested by the final consumer. 

The results obtained in a recent study by Brera *et al.* [39] showed a significant DON reduction from the caryopsis to cooked pasta, accounting for a mean DON contamination decrease of 78%. Moreover, the overall DON reduction observed from wheat grain to dry pasta was 66%.

Another study conducted by Visconti *et al.* concluded that the retention level of DON from grains on the market to cooked pasta on the plate can be conservatively assessed at 25% or less [40]. 

As far as DON bio-accessibility, a recent *in vitro* study has demonstrated differences in levels of DON during the child digestion processes, attributable to different typologies of pasta and initial contamination levels [41].

L. González-Osnaya *et al.* evaluated the occurrence of DON at a rate of 28% in bread whereas in pasta the occurrence was higher, varying from 9.3% to 62.7%. The mean content of deoxynivalenol in bread was 42.5 μg/kg while in pasta the content of deoxynivalenol was higher (137.1 μg/kg). The estimated daily intake of deoxynivalenol from the consumption of the mentioned products represented 8.4% of the tolerable daily intake [42]. 

Bockhorn *et al.* analysed 29 pasta samples purchased from retail shops in Berlin in April and May 2001 and were analysed for their content of deoxynivalenol. Ninety percent of the raw samples contained less than 0.5 mg DON/kg, but three out of 29 samples had contamination of up to 0.84 mg/kg. The amount of DON decreased after cooking, resulting in 60%–80% lower DON levels in the ready to eat products [43]. 

## 2. Results and Discussion

### 2.1. Exposure Assessment

Exposure of different population groups was calculated by a deterministic approach using the following equation:

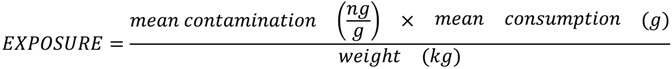
(1)


As far as the calculation of DON intake derived from only pasta, three different parameters were taken into account: the mean contamination value, the mean consumption rate expressed in grams, and the body weight expressed in kg. The exposure was calculated as ng/kg body weight/day. This unit was chosen to compare the resulting values with the TDI of DON that was set by the Scientific Committee for Food at 1000 ng/kg bw/day [44].

### 2.2. Occurrence Values

The statistical description analysis of the obtained results is shown in Table 1 and Table 2. The mean (64.8 µg/kg) and median (35 µg/kg) DON values of all samples were far from the legal limit of 750 µg/kg.

Ninety-nine percent of samples did not exceed the threshold of 50% of the legal limit. A percentage of 78.6% of samples was lower than the limit of quantification (70 µg/kg). Even the DON contamination levels corresponding to the 95th percentile and 99th percentile were around 50% of the legal limit. 

The contamination profile of a subgroup of pasta samples (N = 43), namely short shape typology, more commonly consumed by elderly and children but not corresponding to baby foods, showed slight different values for the mean (101.5 µg/kg) that were higher than the overall mean. All the other values, *i.e.*, median, 95th percentile, 99th percentile and the maximum contamination value, overlapped with the overall scenario.

No cluster contamination was observed for a specific brand and generally the contamination was equally distributed among the different brands. 

The obtained contamination levels generally confirm previous findings cited before. 

**Table 1:** Descriptive statistics of DON contamination in pasta samples.

Parameter	Numerical value
Number of samples	472
Samples <LOQ	371 (78.6%)
Samples ≥LOQ	101 (21.4%)
Mean contamination (µg/kg)	64.8*
Median contamination (µg/kg)	35*
95th percentile of contamination (µg/kg)	239.4
99th percentile of contamination (µg/kg)	337.0
MAX contamination (µg/kg)	385.7

Note: *Mean and median values have been computed assigning to <LOQ results, the value of LOQ/2 = 35 µg/kg.

**Table 2:** Descriptive statistics of DON contamination in small size pasta samples.

Parameter	Numerical value
Number of samples	43
Samples <LOQ	26 (60.5%)
Samples ≥LOQ	17 (39.5%)
Mean contamination (µg/kg)	101.5*
Median contamination (µg/kg)	35*
95th percentile of contamination (µg/kg)	279.6
99th percentile of contamination (µg/kg)	320.9
MAX contamination (µg/kg)	336.4

Note: *Mean and median values have been computed assigning to <LOQ results, the value of LOQ/2 = 35 µg/kg.

### 2.3. Consumption Rate

Mean consumption rates of pasta related to specific subgroups of population were taken by the Italian official reference database published by Leclercq in 2009 [45]. The study was conducted randomly selecting households after geographical stratification of the national territory. Food consumption was assessed on three consecutive days through individual estimated dietary records. The study sample encompassed 3323 subjects (1501 males and 1822 females) aged 0.1 to 97.7 years belonging to 1329 households.

In Table 3, mean, 95th percentile and 99th percentile consumption rates for total population, consumers only, children, adolescents, adults and elderly, are reported. For children, the data are reported combining males and females. *Vice versa*, for adolescents, adults and elderly, a distinction of gender is provided. 

The range of consumption rates is between 54.2 g/d and 161.7 g/d being this latter value the worst in absolute terms since it corresponds to children 3–9.9 years whose body weight is to be considered unfavorable for the intake. 

For this reason, the exposure assessment was calculated considering in more detail the status only for children.

**Table 3:** Mean, 95th percentile* and 99th percentile** of individual daily consumption of pasta in the total population (TP), in consumers only (C) and in males (M) and females (F) of different ages (g/d).

Category		Gender	Consumption (g/day)		
			Mean	95th percentile	99th percentile
Total population			54.2	108.7	140.1
Consumers only			59.5	110.7	141.9
Children (3–9.9 years)	Total population		58.2	104.9	161.7
	Consumers only		59.8	104.9	161.7
Adolescent (10–17.9 years)	Total population	M	63.6	128.0	133.3
		F	56.6	105.3	133.3
	Consumers only	M	66.7	128.0	133.3
		F	61.0	105.3	133.3
Adult (18–64.9 years)	Total population	M	60.3	118.4	156.1
		F	47.7	100.0	134.8
	Consumers only	M	66.0	121.6	156.9
		F	53.8	102.2	137.8
Elderly (≥65 years)	Total population	M	61.1	109.6	129.8
		F	50.7	100.6	117.4
	Consumers only	M	64.3	116.5	131.2
		F	54.5	110.9	121.5

### 2.4. Body Weights

Body weights of children between 3 and 5 years were taken from the WHO official database [46], and between 6–14 years, 15–18 years and over 18 years values as reported from EFSA [47] were considered. In Table 4, the corresponding values are reported.

**Table 4:** Mean weight (kg) in all groups of population between 3 and >18 years.

AGE (years)	Boys	Girls
3	14.3	13.9
4	16.3	16.1
5	18.3	18.2
9.9	31.2	31.9
10–14		45
15–18		60
>18		70

### 2.5. DON Exposure in Adolescents and Adults

In all cases, considering mean DON occurrence levels as a fixed parameter and mean, 95th percentile and 99th percentile consumption rates, the overall exposure of the Italian population sub-groups has to be considered not at risk. For instance, for adolescents, even in the worst case, *i.e.*, 133.3 g/d corresponding to the 99th percentile consumption rate—a body weight of 45 kg corresponding to the subgroup of adolescents aged 10–14 years and a mean DON contamination level of 64.8 μg/kg—the exposure would account for 192 ng/kg bw/day corresponding to almost one fifth of the TDI. By taking as a reference situation the exposure of adolescents, all the other population subgroups—adults and elderly—accounted for an even lower exposure rate considering approximately similar consumption rates and higher body weights. For instance, for adults with a 99th percentile consumption rate of 156.1 g/d, a body weight of 70 kg and considering the DON mean contamination level, the exposure resulted in 144.5 ng/kg bw/day. 

### 2.6. DON Intakes in Children

As far as children, different scenarios for their exposure assessment were taken into consideration consistently with different consumption rates (mean, 95th percentile and 99th percentile) and contamination levels (mean level, threshold level, legal limit) (Table 5). By accounting for the daily mean consumption rate of pasta corresponding to 59.8 g for children aged from 3 to 9.9 years, the exposure, only related to the consumption of pasta, did not reveal any alarming situation, being quite far below the TDI. 

More specifically, the obtained exposure rates decreased from 3 year old children to 10 year old children accounting for a mean value, combining male and female data, of 275 ng/kg bw/day (27% of the TDI) to 123 ng/kg/bw/day (12% of TDI), respectively. 

A second scenario was evaluated taking into consideration double the mean contamination level, *i.e.*, 130 μg/kg; also in this condition, the exposure equalled 50% of the TDI in the worst case (children aged 3 years). No meaningful difference between male (543 ng/kg bw/day) and female (559 ng/kg bw/day) groups was noted. 

A third scenario considering the calculated DON contamination level leading to an exposure corresponding to the TDI for every subgroup of children was also considered. The resulting levels ranged from one third to two third of the legal limit, as set by the Regulation 1126/2007 [25]. 

Nevertheless, it must be considered that 99% of the results obtained from this study fell below these contamination levels. 

In Table 6, the same scenarios were calculated but for consumption rates corresponding to the 99th percentile, *i.e.*, 161.7 grams. Even in this case, the exposure corresponding to the mean occurrence level obtained in the present study always fell below the TDI with a maximum value for the exposure accounting for the 75% of the toxicological threshold in the worst case (females aged 3 years). 

**Table 5:** DON exposure in children (males (M) and females (F)) assuming a mean consumption of pasta of 59.8 g for children aged from 3 to 9.9 years.

DON contamination (µg/kg)	Weight (kg) /age (years) males	Exposure of males (ng/kg bw/day)	Weight (kg) /age (years) females	Exposure of females (ng/kg bw/day)
64.8*	14.3/3	271	13.9/3	279
	16.3/4	237	16.1/4	240
	18.3/5	211	18.2/5	213
	31.2/10	124	31.9/10	121
130	14.3/3	543	13.9/3	559
	16.3/4	476	16.1/4	482
	18.3/5	424	18.2/5	427
	31.2/10	249	31.9/10	243
239 (M)–233 (F)	14.3/3	999	13.9/3	1002
273 (M)–269 (F)	16.3/4	1001	16.1/4	999
306 (M)–305 (F)	18.3/5	999	18.2/5	1002
522 (M)–533 (F)	31.2/10	1000	31.9/10	999

Note: *: DON contamination level (N = 472).

**Table 6:** DON exposure in children (males (M) and females (F)) assuming a 99th percentile consumption of pasta of 161.7 g for children aged from 3 to 9.9 years.

DON contamination (µg/kg)	Weight (kg) /age (years) males	Exposure of males (ng/kg bw/day)	Weight (kg) /age (years) females	Exposure of females (ng/kg bw/day)
64.8*	14.3/3	732	13.9/3	753
	16.3/4	643	16.1/4	650
	18.3/5	572	18.2/5	575
	31.2/10	335	31.9/10	328
89 (M)–86 (F)	14.3/3	1006	13.9/3	1000
101 (M)–100 (F)	16.3/4	1002	16.1/4	1004
114 (M)–113 (F)	18.3/5	1007	18.2/5	1003
193 (M)–198 (F)	31.2/10	1000	31.9/10	1003

Note: *: DON contamination level (N = 472).

Analogously to the previous description of different scenarios, the DON concentration levels leading to an exposure equal to the TDI were calculated resulting in all children subgroups as a higher value than the DON mean contamination level obtained in this study. 

**Figure 2:**
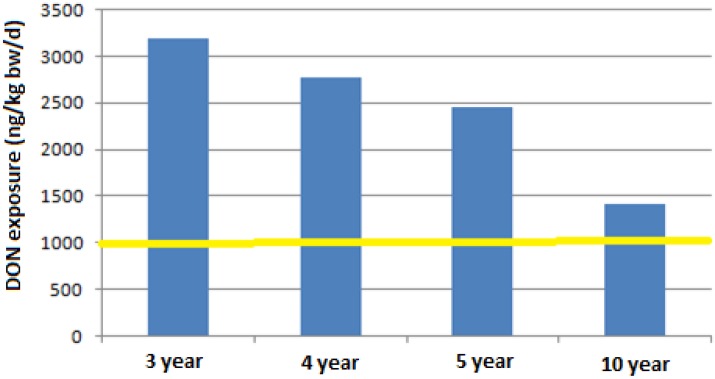
DON exposure for a consumption of pasta of 59.8 g (mean consumption of pasta by children between 3 and 9.9 years old, [45]) at a contamination level of 750 µg/kg.

**Figure 3:**
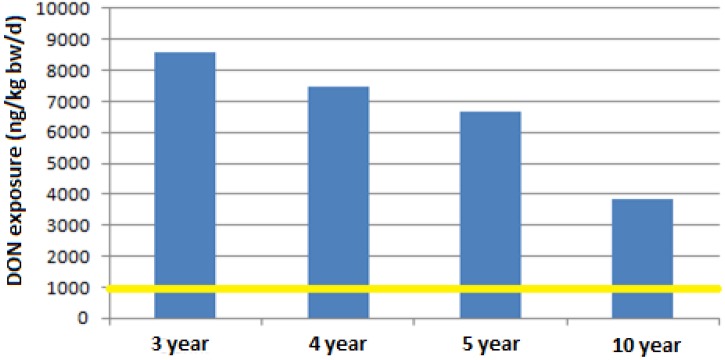
DON exposure for a consumption of pasta of 161.7 g (99th percentile of consumption of children between 3 and 9.9 years old, [45]) at a contamination level of 750 µg/kg.

A last scenario, shown in Figure 2 and Figure 3, leads to a very challenging issue since if the legal limit of 750 μg/kg is considered, for any consumption rate, both mean and high values (99th percentile), and for all children subgroups, an increase of 1.5–8 times the TDI would be reached, respectively.

## 3. Experimental Section

### 3.1. Sampling

In order to assess the level of DON contamination in pasta, 472 packages of commercially produced pasta taken from four large-scale retail traders distributed in different geographical areas in Italy were collected over a period between November 2010 and May 2011. Pasta samples were chosen with the criterion to guarantee the maximum representativeness of the most diffused marketed and consumed products. The selection was chosen on the basis of the market share and consumption rates in the north, south and centre of Italy. Forty-one brands were sampled.

The collected samples had to meet the following characteristics:

- Typology of pasta products (dry, fresh (10%), whole (10%), not egg-pasta, not addressed to baby food chain);
- Random selection among different shape of pasta products (spaghetti, medium size like rigatoni, short size like penne);
- Only one 500 g package per lot, typology, shape and brand.

### 3.2. Principle of the Method

The analytical method used for the analysis of pasta samples got accreditation, number 0779, by the national accreditation body ACCREDIA, for DON determination in wheat and derived products.

DON is extracted from the matrix by homogenizing the sample with water. After centrifugation, the extract is cleaned-up step by immunoaffinity columns and the toxin is eluted with methanol, dried under nitrogen and re-dissolved in an aqueous solution of methanol (9.5%). DON is quantified by reversed-phase HPLC by UV detection.

According to the obtained linearity, the used analytical method for DON determination in pasta samples is to be considered applicable in the range 70 µg/kg ÷ 2000 µg/kg.

### 3.3. Sample Preparation

The first step was to choose between a wet (slurry) and a dry homogenization of pasta samples. The preparation of slurried samples by using a lab-scale 4-lt Waring Blender and adding to the sample tap water in a ratio 1:1, led to very sticky products. For this reason, this approach was abandoned and a processing of samples in dry conditions was preferred. 

All pasta samples were ground with a laboratory mill (Retsch ZM 200, Retsch GmbH, Haan, Germany) to obtain a particle size of at least 0.75 mm. After grinding and before drawing the test aliquot, a thorough homogenization of the test portion was done. 

#### 3.3.1. Extraction

DON is extracted by weighing 20.0 g (±0.1 g) of the dry ground test portion directly in the blender. One hundred and sixty milliliters of deionized water as extraction solvent was added. After 3 min of blending, the extract is centrifuged at 8000 rpm per 10 min.

The extract is then transferred to the IAC.

#### 3.3.2. Immunoaffinity Clean-Up

To clean samples, manufacturers’ instructions of IAC (DONPREP, R-Biopharm-Rhone, Glasgow, UK) were followed. IAC columns were kept at room temperature before the conditioning. 

Under slight vacuum conditions, 3 mL (equivalent to 0.375 grams of pasta) of the centrifuged extract was pipetted into the syringe connected to the IAC. During the clean-up, the flow was maintained at a constant flow of not more than 3 mL/min, (in any case, the flow speed must not exceed 5 mL/min).

After the complete passage of the filtered sample, IAC was washed with 5 mL of de-ionized water. The column was successively dried by flowing at least 2 volumes of air.

DON was eluted from the IAC in two steps: firstly, 750 μL of methanol were flown by gravity and then, after 1 min, a second amount of 750 μL of methanol was applied to the IAC. 

To collect the entire residual methanol, the elution is completed by pushing one volume of air inside the IAC by a 10-mL syringe. 

The eluate is taken to dryness with a nitrogen flow at a temperature of 40 °C. The residue is then redissolved in 750 μL of the injection mixture MeOH:H_2_O (9.5%:90.5%, v:v), and mixed thoroughly by vortex. The sample to be injected was stored at 4 °C until the HPLC analysis. 

### 3.4. HPLC Analysis

One-hundred and fifty microliters (equivalent to 0.075 grams of pasta sample) out of 750 µL of the reconstituted analytical sample were injected onto HPLC, by partial loop.

#### HPLC Operating Conditions

The following operating conditions were used:

- Chromatographic column: Symmetry^®^ C18 (Waters, Milford, Massachusetts, UK) reversed phase, 5 µm, 4.6 mm × 150 mm, kept at constant temperature of 40 °C;
- Mobile phase: deionized water: methanol 85:15 v:v;
- Flow rate: 1.0 mL/min;
- UV spectrophotometer regulated at a wavelength of 220 nm;
- Injection Volume: 150 µL.

### 3.5. Spiking Procedure

To assess the recovery factors to be used for correcting the analytical results, six replicates of fortified samples were prepared by weighing directly in the homogenizer 20.0 g ± 0.1 g of blank pasta samples and pipetting proper volumes of working solution of DON reference standard to obtain spiking concentration levels of 200 µg/Kg and 750 µg/Kg. The fortified samples were left under hood for two hours before the successive processing. 

### 3.6. Validation Study

With the aim to test the reliability of the analytical results, a single-laboratory validation study was performed on the used method according to the IUPAC protocol [48]. 

All method performance characteristics, including precision and accuracy, were compliant with the criteria set by the European Commission Regulation (CE) 401/2006 [49]. 

#### Limit of Quantification

The limit of quantification (LoQ) of the used method was calculated over replicate analyses of blank samples and calculating mean values and standard deviation of the areas of the background. The standard deviation was then multiplied by 10. 

From this approach a LoQ of 70 µg/kg was calculated. In these analyses, a value of repeatability ≤10% was verified. 

### 3.7. Calibration Curve

Fit for purpose calibration curve was built up by preparing six working solutions in 5-mL flasks. Each level was injected in triple. 

The stock solution of the primary certified standard material (Trilogy^®^, R-Biopharm: 100 µg/mL) was diluted with a mixture water:methanol 90.5:9.5 (v:v) to 10 µg/mL, by pipetting 1000µL of it in 10-mL flask. To prepare working solutions necessary for the building up of the calibration curve, proper amounts of DON were drawn from the diluted solution. 

## 4. Conclusions

Estimates of dietary exposure to DON based on the obtained occurrence data in pasta samples are far below the TDI of 1000 ng/kg b.w. for populations of all age groups, and therefore do not represent a health concern.

Just in some cases regarding population subgroups’ exposure to DON, some borderline situations were noted. In fact, considering a DON contamination level slightly higher than the mean value obtained in this study—like for instance 90–100 µg/kg, for children from 3 to 5 years with high consumption rates (99th percentile)—the exposure levels would reach the TDI, considering pasta as the only daily contribution to exposure. 

However, it should be noted that the abovementioned concentration value would correspond to almost one seventh of the legal limit; therefore, creating a quite challenging situation where even for very low levels of DON contamination in a food product highly consumed such as pasta, a health warning for some sensitive consumer groups could be raised.

It should be also noted that, due to the high DON hydro-solubility, the overall exposure assessment should, however, be re-examined considering a loss of up to 20% of DON after cooking pasta, except for when the cooking water remains in the meal as in the case of soups, consumed especially by the elderly and young children.

The scientific information derived from this study can provide a basis for more strict consideration of children’s exposure through diet, since even for contamination levels far lower than the legal limit, borderline situations can arise.

This consideration is more pertinent if applied to DON that is present in the wide spectrum of food products such as bread, breakfast cereals, pizza, biscuits and other cereal-based products.

For the above, reconsideration both of the maximum tolerable legal limit and of the toxicological threshold could represent interesting and challenging topics to be examined with the aim to guarantee an even higher level of safety for those population subgroups, such as children and adolescents, for whom in some cases issues could arise.
